# Supplementary material for: The genome sequence of the Goose Foot Starfish, Anseropoda placenta (Pennant, 1777) (Valvatida: Asterinidae)
Source: Wellcome Open Res. 2025 Oct 24;10:604. [Version 1] doi: 10.12688/wellcomeopenres.25057.1 (PMC12961294; doi:10.12688/wellcomeopenres.25057.1)
Supplement: Peer review history

# Reviewer report: Reviewer response for version 1

**DOI:** 10.21956/wellcomeopenres.27623.r149637

**Published:** 2026-03-18

**Authors:** Jayan Duminda M Senevirathna

**Affiliations:** 1 Uva Wellassa University, Badulla, Uva Province, Sri Lanka

The article is well written and scientifically sound. However, the following minor revisions are suggested to further strengthen the manuscript:

 In Figure 2, the unit of coverage (x) should be clearly indicated on the X-axis to improve clarity and interpretation of the figure.

 In Table 2, the longest scaffold length (Mb) should also be reported for eaAnsPlac2.hap2.1 to maintain completeness and consistency with the other assembly statistics.

 The percentage of the assembly assigned to chromosomes appears to be less than 90% based on table 4, even though haplotype 1 is described as a chromosome-level assembly. The authors should clarify this discrepancy and explain how chromosome-level assembly was determined in this case.

Are sufficient details of methods and materials provided to allow replication by others?

Yes

Is the rationale for creating the dataset(s) clearly described?

Yes

Are the datasets clearly presented in a useable and accessible format?

Yes

Are the protocols appropriate and is the work technically sound?

Yes

Reviewer Expertise:

Aquatic Genomics

I confirm that I have read this submission and believe that I have an appropriate level of expertise to confirm that it is of an acceptable scientific standard.

# Reviewer report: Reviewer response for version 1

**DOI:** 10.21956/wellcomeopenres.27623.r149635

**Published:** 2026-03-04

**Authors:** Jullien Flynn

**Affiliations:** 1 université laval, Quebec City, Canada

In this article, the authors present a diploid genome assembly for *Aneropoda placenta *using PacBio Hifi sequencing and HiC scaffolding.  One haplotype of the assembly placed the sequences into 22 major scaffolds which likely represent 22 chromosomes. The authors evaluate various metrics of the assembly and make the data and methods publicly available. This article meets the standards of the field.

Are sufficient details of methods and materials provided to allow replication by others?

Yes

Is the rationale for creating the dataset(s) clearly described?

Yes

Are the datasets clearly presented in a useable and accessible format?

Yes

Are the protocols appropriate and is the work technically sound?

Yes

Reviewer Expertise:

Genomics, repetitive DNA, developmental biology in Drosophila

I confirm that I have read this submission and believe that I have an appropriate level of expertise to confirm that it is of an acceptable scientific standard.

# Reviewer report: Reviewer response for version 1

**DOI:** 10.21956/wellcomeopenres.27623.r148125

**Published:** 2026-03-04

**Authors:** Yichen Dai

**Affiliations:** 1 Fudan University, Shanghai, China

This paper describes a detailed report of a genome assembly from an individual *Anseropoda placenta* (Goose Foot Starfish). A full and detailed account of how the individual used for genome sequencing was collected was provided, along with a clear and systematic description of how DNA was extracted and how the genome was assembled is provided. This genome is the first high-quality genome for the genus *Anseropoda* and also provides valuable data for researchers interested in animal evolution. 

 My only suggestion for the authors would be to provide a brief phylogenetic tree to indicate where the genus *Anseropoda* is in relationship to other starfish species, or to cite a paper with a clear phylogeny of starfish species, just so the readers can have a better concept when reading the introduction section. I think this paper can be indexed in its current form, and no other changes are required.

Are sufficient details of methods and materials provided to allow replication by others?

Yes

Is the rationale for creating the dataset(s) clearly described?

Yes

Are the datasets clearly presented in a useable and accessible format?

Yes

Are the protocols appropriate and is the work technically sound?

Yes

Reviewer Expertise:

Animal evolution; Genome sequencing

I confirm that I have read this submission and believe that I have an appropriate level of expertise to confirm that it is of an acceptable scientific standard.
